# Physician altruism under the change from pure payment system to mixed payment schemes: experimental evidence

**DOI:** 10.1186/s12913-023-09112-4

**Published:** 2023-02-02

**Authors:** Yue Zhang, Xing Li, Xinyuan Zhang, Xinyan Li, Xing Lin, Youli Han

**Affiliations:** School of Public Health, Capital Medical University, No.10 Xitoutiao, Youanmenwai Street, Fengtai District, Beijing, 100069 China

**Keywords:** Altruism, Diagnosis-related groups, Fee-for-service, Mixed payment schemes, Laboratory experiment

## Abstract

**Background:**

Mixed payment schemes have become one of the effective measures to balance medical costs and quality of medical services. However, altruism as an intrinsic motivation may influence the effect of switching from a pure payment system to mixed payment schemes. This study aimed to quantify physicians’ altruism and analyze the effect of changes of payment system on physicians’ altruism and thus proposed references for the reform of payment system.

**Methods:**

We simulated an exogenous payment system in a controlled laboratory with five experimental groups and 150 medical student subjects. Physicians’ altruism was measured by estimating altruistic parameter and marginal rate of substitution. The non-parametric test and the least square regression analysis were used to analyze the differences of altruistic parameters between pure payment systems and mixed payment schemes. Finally, we analyzed the effect of changes in payment system accompanied by changes in trade-off range on physicians’ altruism.

**Results:**

We find that the mean value of individual altruistic parameter is 0.78 and the marginal rate of substitution is 1.078. Their estimates at the individual level were significantly positively correlated (Spearman’s *ρ* = 0.715, p < 0.01). The shift from pure payment system to mixed payment scheme reduced the altruistic parameter. However, the altruistic parameter increased with the increase of the trade-off range. Physicians who were more altruistic generated higher patients’ health benefit. For each unit increase in altruistic parameter, the increase in patients’ health benefit was lower in mixed payment scheme than in the pure payment system.

**Conclusion:**

The estimates of altruistic parameters are reliable. Physicians attach a higher weight to patients’ benefit than to their own profit. Mixed payment schemes improve physicians’ behavior and relate to lower altruistic parameters; physicians only need to sacrifice less personal profits to generate the same or even higher altruistic parameter as under the pure payment system. The design of mixed payment schemes that make the interests of physicians and patients close to each other by reducing the trade-off range can provide implication for the reform of payment system in which the physicians’ interest and the patients’ benefit are consistent.

**Supplementary Information:**

The online version contains supplementary material available at 10.1186/s12913-023-09112-4.

## Background

With the establishment of universal healthcare coverage, controlling the rapid rise of medical costs and guaranteeing the quality of medical services have become important challenges. The reform of the medical insurance payment system is one of the effective measures to balance costs and quality of medical services. Medical insurance compensation in form of pure payment systems as a main source of income for medical service providers can be divided into retrospective payment and prospective payment, depending on the timing of determination of the payment rate. However, there are some shortcomings in pure payment systems. Specifically, the retrospective payment is designed to compensate service providers for their cost and may lead to an oversupply. The prospective payment aims to establish a risk-sharing mechanism between payers and service providers, which may lead to an undersupply. Mixed payment schemes can offset some deficiencies of these two pure payment systems and get closer to a balance between cost compensation and risk sharing. Since 2016, China has proposed the implementation of multivariate composite medical insurance payment systems and encouraged the implementation of diagnosis-related groups (DRG).

The guiding and restraining effects of the change in payment systems largely depend on the behavioral response of medical service providers. However, altruism as an intrinsic motivation may influence physicians’ behavioral responses. Physicians’ altruism is implicit in statements about medical professional values [1]. It is the key to ensure the welfare of patients. The information asymmetry between physicians and patients, the uncertainty of medical treatments and health outcomes, are two distinct characteristics of healthcare that make patients unable to judge the quality of services before receiving them and thus become heavily dependent on physicians. Meanwhile, physicians inevitably have to balance between their own interest and patients’ benefit due to the limitation of medical resources. Additionally, the principal–agent relationship between physicians and patients and the resulting moral hazard make it difficult to design an optimal contract to effectively govern physicians’ behavior [2, 3]. Therefore, people hope that physicians can pay more attention to patients’ benefit and fully reflect their altruism in healthcare.

Altruism is generally defined in economics as a deviation from purely self-interested behavior to benefit others at one’s own expense. In health economics, altruism is embedded in the physician–patient relationship and defined as physicians’ weight on patients’ health benefit in the utility function [4]. The importance of physicians’ altruism has been emphasized in theoretical studies, especially for the design of payment systems. Since Arrow [5] highlighted the importance of physicians’ benevolent motivation, the altruistic-physician assumption has become quite common in modeling physicians’ behavior [2, 6–9]. Ellis and McGuire [7] modeled physicians as deriving utility from both their own profit and patients’ benefit, which leads to important implications for the design of optimal payment schemes. Chalkley and Malcomson [6] proposed that the optimal cost-sharing rate depends on the extent to which the physician considers the patients’ health welfare. When the physician’s altruism is unknown, incentive mechanisms need to be designed to reveal it [9]. Some theoretical studies suggest the importance of quantifying physician altruism for payment system and incentive mechanism design [2, 9, 10]. When the degree of altruism differs among physicians, some scholars modeled the impact of pay for performance (P4P) on the provision of medical service and physicians’ treatments and referral decisions [10, 11]. Liu and Ma [12] studied the delegation of treatment plans; they found that the first-best of physicians with different levels of altruism depended on whether physicians can commit to treatment plans at the time of contract acceptance.

Despite the theoretical literature having highlighted the importance of altruism, few empirical studies have successfully quantified the physicians’ altruism given the complexities in medical decision-making and the resulting challenges in quantifying physicians’ profit and patients’ benefit using field data [4]. They are mainly based on the theory of revealed preference [13] and random utility theory [14–16]. The theory of revealed preference is based on the utility maximization, rational choice, and stable preference. It holds that the choices selected by individuals according to their preferences are necessarily utility maximization, and the utility maximization is revealed by individuals’ choices. The main methods are strategic games, such as dictator game (DG) and ultimatum game. Brosig-Koch et al. [17] measured physicians’ altruism through laboratory experiments simulating DG in medical decision-making scenarios; their results showed that altruism is heterogeneous and plays a role in service provision, and it can partially mitigate agency problems. Li [18] and Li et al. [19] used a modified DG to reveal individual’s altruism. They proved that altruism is heterogeneous and can predict students’ expected specialty choice and practicing in an underserved area. However, the random utility theory argues that selection is uncertain [14], and that individuals often make different choices when faced with the same options repeatedly [20–23]. Therefore, some scholars have proposed to add a random component to the traditional utility function [16, 24, 25]. Godager and Wiesen [26] used a random utility model to estimate physicians’ altruism; they observed a variation in physician altruism and presented that a ‘one size fits all’ payment scheme cannot implement the first-best medical service volume.

The design of a payment system depends on the situation of physicians’ altruism; thus revealing and measuring physicians’ altruism is the premise and key to optimize the design of a payment system. Inspired by Brosig-Koch et al. [17], in this study, we designed a controlled laboratory experiment of a payment system in the form of DG in the context of medical decision-making to induce physicians’ altruism. The payment system included both pure payment systems and mixed payment schemes, because the former with strong economic incentive may crowd out the altruistic intrinsic motivation [27, 28]. Such a design allowed us to compare the altruism of physicians under the change in payment systems. The shift from pure payment systems to mixed payment schemes has been emphasized in some theoretical and empirical studies [7, 29–31]. Through economic game theory, the study of Qing et al. [32] indicated that choosing a mixed payment strategy according to a certain probability distribution can always achieve the Pareto equilibrium solution of resource allocation and can also be helpful to control excessive growth of medical expenses. Evidence from a natural experiment conducted by Dumont et al. [33] showed that mixed compensation schemes reduce physician service quantity and increase their average time spent per service relative to fee-for-service (FFS). Brosig-Koch et al. [17, 34, 35] compared the effects of pure payment systems and mixed payment schemes on physicians’ behavior under controlled laboratory conditions. They found overprovision in FFS and underprovision in capitation (CAP), both of which could be reduced by mixed payment schemes. In mixed-FFS (mixed-CAP) schemes, a higher share of CAP (FFS) leads to further reduction in overprovision (underprovision). Moreover, mixed payment schemes generally provide a higher benefit–remuneration ratio than the respective pure payment systems. Green [28] explored how six prominent payment systems influenced physicians’ behavior in a laboratory experiment. The results showed that the retrospective payment systems (FFS and FFS with P4P) resulted in the lowest overall quality of services, while the prospective payment systems (salary [SAL], CAP, CAP with report card, and CAP with P4P) performed better.

In summary, more countries are using mixed payment schemes instead of pure payment systems to continuously optimize the quantity and quality of services delivered [36–38]. Several studies have discussed the measurement of altruism and its implications for the design of payment systems, and some discussed the effects of FFS, CAP, P4P, and the corresponding mixed payment schemes on physicians’ behavior, but few have focused on their effect on physicians’ altruism. Therefore, we aimed to quantify physicians’ altruism and explore the impact of the change in payment systems on physicians’ altruism through a controlled laboratory experiment to provide a reference for the reform of the payment system while considering the effects of physicians’ altruism.

## Methods

### Experimental design

Based on the model of Ellis and McGuire [7], as well as the experiment by Brosig-Koch et al. [17], we designed a controlled laboratory experiment to explore, ceteris paribus, the effects of switching from a pure payment system to mixed payment schemes on physicians’ altruism. The Eqs. (1) and (2) of physicians’ remuneration *R*(*q*) and profit *π*(*q*) and the Eq. (3) of patients’ health benefits *B*(*q*) were consistent with Brosig-Koch et al. [17]. In our experiment, medical students played the role of physicians and chose the quantity of medical service* q* $\in$ [0, 10] for nine different patients *j* $\in$ [1, 9] with three illnesses *k* $\in$ [*A*, *B*, *C*] and three severities *l* $\in$ [*x*, *y*, *z*]. If physician *n* provided *q* for *j*, *R*(*q*), *π*(*q*), and *B*(*q*) were determined as follows:

1 $$R\left(q\right)=\mu L+(1-\mu )pq$$

2 $$\pi \left(q\right)=R(q)-c(q)$$

3 $$B\left(q\right)=\left\{\begin{array}{c}{B}_{0}+\theta q, q\le {q}^{*}\\ {B}_{1}-\theta q, q\ge {q}^{*}\end{array}\right.$$

*L* is a lump-sum payment per patient; *p* is a fee per service; *μ* $\in$ [0, 1] is the weight on the lump-sum component. *c*(*q*) is the cost per service; *c*(*q*) = 0.1·*q*^2^ [2, 30]. *B*_0_ is the patient’s initial state of health; *θ* is the marginal health benefit (a certain change of health benefit induced by an additional unit of medical service); q^*^ is the quantity chosen that maximized *B*(*q*); *B*_1_ = *B*_0_ + 2θq^*^. In order to compare the impact of the change in payment system on physicians’ altruism, we used a within-subject design, which means subjects had to participate in two parts of the experiment: Part I was incentivized by non-blended FFS or non-blended DRG, and Part II by mixed payment schemes, which is a mixture of DRG and FFS in different proportions. The design of FFS, DRG, and a mixture of the two, as well as the parametric of *R*(*q*), *π*(*q*), *c*(*q*) and *B*(*q*) are detailed in our recently published article [39]. Although the stylized decision setting abstracts from the complexity of real-world physicians’ decision-making, it can isolate the trade-off between the physicians’ profit and the patients’ health benefit and still inherit the incentives occurring in a real-world setting [26].

The experiment involved seven groups (see [39]), but this study only focused on the first five groups. They are three groups of DRG and mixed-DRG payment schemes (Mix-DRG-2, Mix-DRG-4 and Mix-DRG-6) and two groups of FFS and mixed-FFS payment schemes (Mix-FFS-6 and Mix-FFS-8). The weight of DRG in *y* was set to 0.96, 0.84 and 0.64 in Mix-DRG-2, Mix-DRG-4 and Mix-DRG-6 and that in *x* (*z*) was 0.97 (0.95), 0.85 (0.83) and 0.65 (0.63). The weight of FFS in *y* was set to 0.6 and 0.8 in Mix-FFS-6 and Mix-FFS-8 and that in *x* (*z*) was 0.59 (0.61) and 0.79 (0.81). They were chosen because their incentive intensity has changed substantially (*q*ˆ–the *q* chosen that maximized *π*(*q*)–is not equal in the pure payment system and mixed payment schemes).

### Experimental protocol

The computerized experiment programmed with z-Tree [40] was conducted at the Laboratory for Experimental Economics at the Capital Medical University. Overall, 150 medical students were recruited through online posters, and they were randomly assigned to one of five experimental groups of 30 people each. There were 85 undergraduates (third year and above), 65 graduate students, and 106 female students. The average age of the included students was 22 years. There was no difference in the distribution of age, education and gender among different groups (*p* ≥ 0.11). Each group participated in Part I (pure payment system) and Part II (mixed payment schemes) in sequence. The subjects in each part decided the *q* for each of the nine patients. Each experiment was conducted for five rounds. The instructions for the experiment were in the additional file 1 and the screen shot of the decision-making presented physicians’ choice menus was in the additional file 2.

### Quantification of altruism

Physicians acting as agents of patients and hospitals to make decisions, weighing personal (hospital) benefits and patients’ health benefits, and physicians’ preference for patients’ health benefits reflects their altruistic behavior. Altruism is the degree to which utility-maximizing physicians attach importance to *B*(*q*) in the trade-off between *π*(*q*) and *B*(*q*). The utility function of physicians is aligned with Brosig-Koch et al. [17]. Suppose physician *n* chooses the *q* to maximize their personal utility:

4 $${U}_{n}\left(q\right)=(1-{\alpha }_{n})\pi (q)+{\alpha }_{n}B(q)$$

*α* $\in$ [0, 1], which is a measure of individual altruism; the larger the *α*, the higher the altruism; *α* = 0 represents a purely profit-maximizing physician; *α* = 1 represents a purely altruistic physician. Combined with the Eqs. (1), (2), (3), and (4), *α* can be calculated by using the first derivative of the utility function equal to zero; *U*^ʹ^(*q*) = 0. For *q* ≤ q^*^, *U*^ʹ^(*q*) = *α*[*θ* − (1 − *μ*)*p* + 2∙0.1∙*q*] + (1 − *μ*)*p* − 2∙0.1∙*q*; *U*^ʹ^(*q*) = 0, *α* = [2∙0.1∙*q* − (1 − *μ*)*p*] / [2∙0.1∙*q* − (1 − *μ*)*p* + *θ*]; *q* ≥ q^*^, *U*^ʹ^(*q*) = *α*[− *θ* − (1 − *μ*)*p* + 2∙0.1∙*q*] + (1 − *μ*)*p* − 2∙0.1∙*q*; *U*^ʹ^(*q*) = 0, *α* = [2∙0.1∙*q* − (1 − *μ*)*p*] / [2∙0.1∙*q* − (1 − *μ*)*p* − *θ*]. Taking *B*_*y*_ under pure FFS as an example, q^*^ = 5, *μ* = 0, and *θ* = 1. When physicians choose *q* = 8, *α* = [2∙0.1∙8 − 1∙2] / [2∙0.1∙8 − 1∙2 − 1] = 0.285. If *q* = 5, *α* = [2∙0.1∙5 − 1∙2] / [2∙0.1∙5 − 1∙2 − 1] = 0.5. In Mix-FFS-8, if *q* = 8, *α* = [2∙0.1∙8 − 0.8∙2] / [2∙0.1∙8 − 0.8∙2 − 1] = 0. If *q* = 5, *α* = [2∙0.1∙5 − 0.8∙2] / [2∙0.1∙5 − 0.8∙2 − 1] = 0.375. More details are shown in additional file 3. To ensure that *α* = 1 when physicians select q^*^, we performed a standardization, namely *α* divided by the *α*^*opt*^ corresponding to the q^*^ for the same patient. This allowed us to compare the differences of individual *α*’s between different payment systems. We calculated the individual *α*’s only for subjects who chose two-thirds or more of the Pareto-efficient *q*, which is the medical service quantity choice in the range between q^*^ and *q*ˆ. The subjects needed to make at least 30 (45∙2/3) Pareto-efficient decisions in Part I and Part II separately, and the individual *α* was the mean value of the *α*’s in two parts.

The above utility function based on the theory of revealed preference is deterministic and does not contain random components. However, when choice is stochastic, the revealed preference analysis often relies on the random utility model [41]. That is, *U*_*ni*_ = *V*_*ni*_ + *ε*_*ni*_ = *β*_*0*_ + *β*_*1*_*X*_*1*_ + … + *β*_*m*_*X*_*m*_ + *ε*_*ni*_, where *V*_*ni*_ represents the utility of the deterministic component, also known as representative utility, which can be explained by observation factor *X*_*m*_. *β*_*m*_ is the weight of *X*_*m*_, whose statistical significance indicates whether it will affect the utility, and the sign and size indicate the direction and degree of the influence on the utility. *ε*_*ni*_ is the random component, which is a function of unobserved factors and individual-level variation in tastes. The utility is a latent variable that is not directly observed, and therefore, the *β*_*m*_ cannot be estimated directly. In a probabilistic framework, when individual *n* is presented with a pair of choices, the probability that *n* chooses *i* over *j* can be written as *P*_*ni*_ = *Pr*(*U*_*ni*_ > *U*_*nj*_) = *Pr*(*V*_*ni*_ + *ε*_*ni*_ > *V*_*nj*_ + *ε*_*nj*_) = *Pr*(*ε*_*ni*_—*ε*_*nj*_ > *V*_*nj*_—*V*_*ni*_). Assuming that *ε*_*ni*_ is an independently and identically distributed extreme value, *β*_*m*_ can be estimated by fitting a logit model. The *β*_*m*_ can then be used to estimate the marginal rate of substitution (MRS), such as the MRS between *B*(*q*) and *π*(*q*), which can be used to estimate physicians’ altruism.

Specifically, physicians’ choice of *q* for each patient can be regarded as a choice set, and the value of *q* ranging from 0 to 10 can be understood as 11 alternatives, each of which contains two attributes of *π*(*q*) and *B*(*q*). The utility function is *U*_*njk*_ = *β*_0_ + *β*_1_*π*(*q*) + *β*_2_*B*(*q*) + *ε*_*njk*_, which is the utility of physician *n* choosing alternative *j* for patient *k*. Therein, the weight on *π*(*q*) was *β*_1_, and that on *B*(*q*), was *β*_2_. They can be estimated by fitting a mixed logit model. The MRS between *B*(*q*) and *π*(*q*) is $-{\beta }_{2}/{\beta }_{1}$, which can be computed by means of the program by Hole [42]. The degree of altruism can be judged by comparing MRS and 1. If MRS is greater than 1, physicians attach a higher weight to *B*(*q*) than to *π*(*q*); if MRS is equal to 1, physicians attach an equal weight to *B*(*q*) and *π*(*q*); if MRS is less than 1, physicians attach a higher weight to *π*(*q*) than to *B*(*q*); the larger the MRS, the higher the altruism.

### Statistical analyses

We explored the differences in physicians’ altruism through non-parametric analysis. Specifically, the Wilcoxon signed-rank (WSR) test was used for comparisons within group, whereas the Mann–Whitney *U* (MWU) test and the Kruskal–Wallis *H* (KWH) test were used for comparisons between groups. Two ordinary least squares (OLS) regression analyses were used to test for the effects of the change in payment system on physicians’ altruism and behavior. The first was ${Y}_{n\left|d\right|}={\beta }_{0}+{\beta }_{1}{Payment}_{(DRG, \, FFS)}+{\beta }_{2}k+{\beta }_{3}l+\lambda {Z}_{n}+{\varepsilon }_{n}$, where $\left|d\right|=\left|q-{q}^{*}\right|$ is the absolute value of deviation in the quantity of medical service provided by physician *n*, ${Payment}_{(DRG, \, FFS)}$ is a set of dummy variables for DRG and FFS payment system, *k* and *l* are type of illness and severity of illness, *Z*_*n*_ is a vector of individual characteristics, and *ε*_*n*_ is an error term. The second was ${Y}_{n\alpha }={\beta }_{0}+{\beta }_{1}{Payment}_{(DRG, \, FFS)}+{\beta }_{2}\left|s\right|+{\beta }_{3}k+{\beta }_{4}l+\lambda {Z}_{n}+{\varepsilon }_{n}$, where *α* is the individual *n*’s altruistic parameter, $| s | = | q^- q*|$is the absolute value of trade-off range. *ε*_*n*_ is assumed to be normal with mean 0 and variance ${\sigma }_{\varepsilon }^{2}$. Tobit regression was used for robustness test.

## Results

### Physicians’ provision behavior

Applying the comparisons within and between groups, we found significant differences in the behavioral responses of physicians to the change in payment system. The $\left|d\right|$ was larger in DRG than in FFS (1.30 [s.d. 1.09] vs. 0.96 [s.d. 0.95], *p* < 0.001, two-sided MWU test). The employment of mixed payment schemes improved physicians’ behavior. In the mixed-DRG schemes, the $\left|d\right|$ decreased to 0.62 (*p* < 0.001, matched-pairs WSR test); in the mixed-FFS schemes, the $\left|d\right|$ decreased to 0.69 (*p* < 0.001, matched-pairs WSR test). In addition to the improved $\left|d\right|$, the ratio of optimal decisions (*r*) and *π*(*q*) also increased. Specifically, from the pure DRG (FFS) to mixed-DRG (mixed-FFS), *π*(*q*) increased from 8.28 (7.98) to 9.62 (9.50); and *r* increased from 0.26 (0.40) to 0.53 (0.50) (*p* < 0.001, matched-pairs WSR test), respectively. In comparisons of the increase of *r* of different *l* (*x*, *y*, *z*) between the pure payment system and mixed payment schemes, we found similar results to the improvement of quantity in our published paper [39]; namely, for *x*, *y,* and *z*, the *r* increased from 0.26 to 0.64 (Mix-DRG-4, *p* < 0.001, matched-pairs WSR test), 0.23 to 0.79 (Mix-DRG-6, *p* < 0.001, matched-pairs WSR test), and 0.61 to 0.78 (Mix-FFS-8, *p* = 0.001, matched-pairs WSR test), respectively. They are detailed in Table 1.

**Table 1:** Changes in indicators of physician behavior and altruism

Conditions	Pure payment systems					Mixed payment schemes				
	$\left|d\right|$	*π*(*q*)	*r*	*α*	MRS	$\left|d\right|$	*π*(*q*)	*r*	*α*	MRS
Mix-DRG-2	1.29	8.24	0.30	0.79	1.08	0.89	9.27	0.42	0.70	0.67
Mix-DRG-4	1.51	8.45	0.22	0.76	0.89	0.58	9.80	0.53	0.68	0.32
Mix-DRG-6	1.10	8.16	0.27	0.83	1.18	0.40	9.77	0.66	0.79	0.34
Mix-FFS-8	0.91	8.02	0.39	0.88	1.13	0.76	9.25	0.47	0.82	0.56
Mix-FFS-6	1.01	7.93	0.40	0.86	1.16	0.63	9.75	0.53	0.68	1.05

OLS regression analysis was used to infer the causal effects of the change in physicians’ behavior. Panel A and panel B in Table 2 show the results for DRG and FFS payment systems. In our model, the dependent variable was $\left|d\right|$. The mixed payment schemes were treated as dummy variables, and the reference categories were pure DRG and pure FFS. We additionally controlled for *k* (*A*, *B*, *C*) and *l* (*x*, *y*, *z*) for *A* and *x*, which were the reference categories, as well as intern experience and gender to investigate the effects of patients’ characteristics and subjects’ characteristics. The results further supported that the deviation was significantly reduced under mixed payment schemes compared with the pure payment system. In the mixed-DRG payment system, underprovision was reduced by about 0.4, 0.7, and 0.9 units under Mix-DRG-2, Mix-DRG-4, and Mix-DRG-6, respectively. Overprovision was reduced by about 0.2 and 0.3 units under Mix-FFS-8 and Mix-FFS-6. The degree of reduced deviation in mixed-DRG (mixed-FFS) increased with the decrease of the DRG (FFS) component. After controlling for *k* (*A*, *B*, *C*) and *l* (*x*, *y*, *z*), the improvement effect of mixed payment schemes remained significant. Compared with *A*, the deviation of *B* and *C* increased in the FFS payment system. Using *x* as the reference category, with the increase of severity, underprovision (oversupply) in DRG (FFS) payment system increased (decreased). Postgraduates with internship experience had less deviation from the quantity of service compared with undergraduates, but it was not statistically significant under FFS payment systems. Compared with male students, the deviation of quantity among female students decreased (increased) under DRG (FFS) payment systems. The purely selfish behavior is reflected in that the subjects always choose the quantity that maximizes personal profit, that is, they choose 0 (10) under a pure DRG (FFS); 2, 4, and 6 (8, 6) under Mix-DRG-2, Mix-DRG-4, and Mix-DRG-6 (Mix-FFS-8, Mix-FFS-6); and the corresponding estimated coefficient of mixed payment schemes was − 2, − 4, and − 6, (− 2, − 4). Our estimated coefficients in DRG (FFS) payment system were not consistent with − 2, − 4, and − 6, (− 2, − 4), so the purely selfish behavior and income effect were not obvious. Tobit regression was used to conduct robustness tests and yielded similar results (see additional file 3).

**Table 2:** OLS regression of $\left|d\right|$

Variable	A.DRG			B.FFS		
	1	2	3	4	5	6
Mix-DRG-2	− 0.412^***^ (0.065)	− 0.412^***^ (0.062)	− 0.392^***^ (0.062)			
Mix-DRG-4	− 0.717^***^ (0.054)	− 0.717^***^ (0.052)	− 0.749^***^ (0.053)			
Mix-DRG-6	− 0.897^***^ (0.051)	− 0.897^***^ (0.053)	− 0.885^***^ (0.052)			
Mix-FFS-8				− 0.207^***^ (0.068)	− 0.207^**^ (0.062)	− 0.192^***^ (0.062)
Mix-FFS-6				− 0.333^***^ (0.063)	− 0.333^***^ (0.060)	− 0.348^***^ (0.061)
*B*		0.041 (0.055)	0.041 (0.054)		0.199^***^ (0.061)	0.199^***^ (0.061)
*C*		− 0.078 (0.054)	− 0.078 (0.053)		0.180^***^ (0.060)	0.180^***^ (0.060)
*y* (intermediate)		0.129^***^ (0.044)	0.129^***^ (0.043)		− 0.434^***^ (0.069)	− 0.434^***^ (0.069)
*z* (severe)		0.370^***^ (0.058)	0.370^***^ (0.057)		− 0.759^***^ (0.066)	− 0.759^***^ (0.066)
Intern experience			− 0.240^***^ (0.044)			− 0.085 (0.052)
Female			− 0.220^***^ (0.053)			0.172^***^ (0.054)
Constant	1.300^***^ (0.038)	1.146^***^ (0.048)	1.399^***^ (0.063)	0.963^***^ (0.041)	1.234^***^ (0.068)	1.142^***^ (0.075)
Observations	1620	1620	1620	1080	1080	1080
*N*	90	90	90	60	60	60
*R*^2^	0.139	0.166	0.194	0.025	0.153	0.160

Robust standard errors are in parentheses

^***^p < 0.01

^**^p < 0.05

^*^p < 0.1

### Physician altruism

**Fig. 1:**
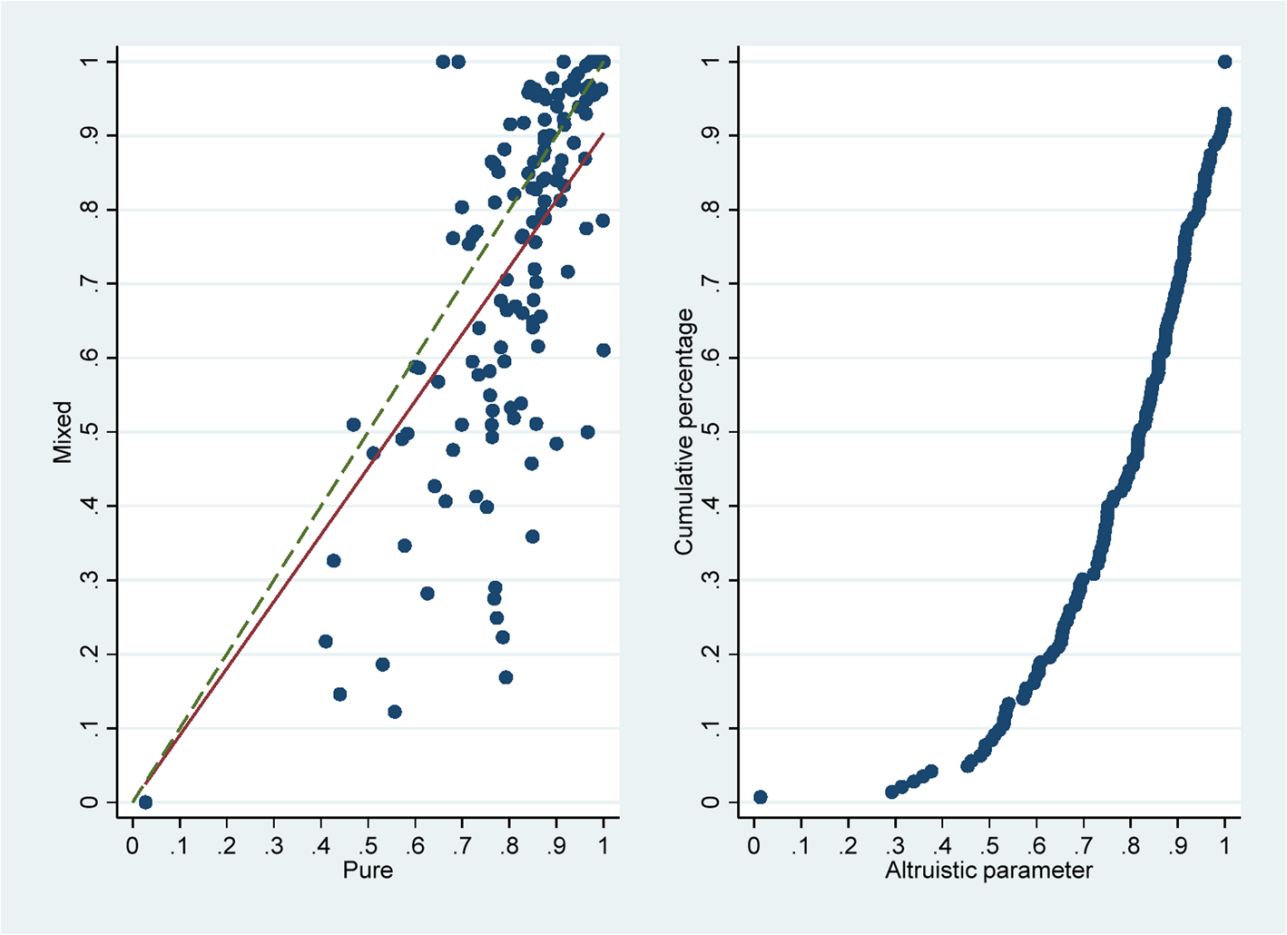
The left panel shows a scatter plot of *α* for subjects in the pure payment system and mixed payment schemes. The right panel shows a cumulative frequency distribution graph of *α*

We calculated individual altruism in the pure payment system and mixed payment scheme. 95.3% (143/150) of the subjects chose two-thirds or more Pareto-efficient quantity of medical service, and they attached a positive weight to *B*(*q*). The scatter plot in the left panel of Fig. 1 depicts the individual *α*’s in the pure payment system (horizontal axis) and mixed payment scheme (vertical axis). The linear fitting line is below the 45-degree line, indicating that the *α* under the mixed payment scheme is lower than that under the pure payment system. The changes of *α* in Table 1 also show that the *α* decrease in general when switching from the pure payment system to mixed payment scheme; the mean value of *α* in the pure payment system and mixed payment scheme are 0.82 (s.d. 0.15) and 0.74 (s.d. 0.24), respectively. However, there was a significant positive correlation between the *α* of the two parts (Spearman’s *ρ* = 0.742, *p* < 0.01). We took the mean value of *α* of the two parts as the individual’s altruism. Overall, the average *α* of the 143 included subjects was 0.78 (s.d. 0.18). The cumulative frequency distribution graph of *α* shown in the right panel of Fig. 1 illustrates that there was substantial heterogeneity in *α*. The *α* was larger than 0.5 for about 90% of the subjects; 50% of the subjects had an *α* above 0.8, and 30% had *α* larger than 0.9.

We further tested the robustness of the *α* by estimating the MRS between *B*(*q*) and *π*(*q*) based on the study of Godager and Wiesen [26]. The MRS is estimated by fitting a mixed logit model. First, the KWH test was used to analyze the differences between groups of each subject’s five rounds of decisions, and the results showed that only five subjects had differences between groups (*p* < 0.05). For them, pairwise comparison was further conducted between groups. After excluding the round with differences from most other rounds, one round was randomly selected from the remaining rounds. For others, one round was randomly selected from the five rounds of decisions. The estimated results showed that the change of MRS is consistent with the decrease of *α* from the pure payment system to mixed payment scheme (Table 1). The MRS under the pure payment system and mixed payment scheme are 1.078 and 0.532, respectively. In order to compare with Godager and Wiesen [26], we took the MRS under the pure payment system as the overall MRS. The details of MRS appear in Table 3. The estimated coefficients showed that *B*(*q*) had a greater impact on physicians’ decision-making. The MRS was larger than 1, indicating that physicians attached a higher weight to *B*(*q*) than to *π*(*q*). The estimation of MRS at the individual level showed that the estimated coefficients of *π*(*q*) for 13 subjects tended to 0, and therefore the MRS could not be estimated. Among the remaining subjects, there was substantial heterogeneity in MRS, with 61 subjects having MRS greater than 1, accounting for about 44.5% (61/137). There was a significant positive correlation between MRS and *α* (Spearman’s *ρ* = 0.715, *p* < 0.01).

**Table 3:** Estimation results from mixed logit model

Variable	β (SE)	SD (SE)	MRS (95% CI)
*π*(*q*)	1.026^***^ (0.070)	0.467^***^ (0.049)	
*B*(*q*)	1.106^***^ (0.060)	0.513^***^ (0.056)	1.078 (0.956, 1.223)
*N*	150		
Observations	14,850		
Log likelihood	− 1984.5721		
LR chi-square	406.65		
Prob > chi-square	< 0.0001		
Akaike criterion	3977.144		
Schwarz criterion	4007.567		

*SE* standard error, *SD* standard deviation, *CI* confidence interval, *MRS* marginal rate of substitution. The 95% CI of MRS was estimated by Krinsky Robb (parametric bootstrap)

***p < 0.001

Based on the robust test of the *α* and their changes, the regression analysis is used to infer the changes of the *α*. We specified the independent and control variables in Table 2 and the trade-off range variable in the regression model (see Table 4). In the DRG payment system, with pure DRG as the reference category, *α* decreased in Mix-DRG-2 and Mix-DRG-4. When the trade-off range was controlled, the increase of the trade-off range was conducive to the increase of *α*, and the decreasing effect of the above two mixed payment systems was no longer significant, whereas *α* increased in Mix-DRG-6. The *k* (*A*, *B*, *C*) and *l* (*x*, *y*, *z*) were further controlled. Taking *x* as the reference category, *y* and *z* increased, and the decreasing effect of Mix-DRG-2 and the increasing effect of Mix-DRG-6 and the trade-off range were still significant. In the FFS payment system, taking pure FFS as the reference category, *α* decreased in Mix-FFS-8 and Mix-FFS-6, and this decreasing effect in Mix-FFS-6 was significant under all control conditions. Postgraduates with intern experience had higher *α* than undergraduates. Female students showed higher (lower) *α* under DRG (FFS) payment systems compared with male students. Tobit regression was further used to conduct robustness tests and yielded similar results (see additional file 3).

**Table 4:** OLS regression of *α*

Variable	A. DRG				B. FFS			
	1	2	3	4	5	6	7	8
Mix-DRG-2	− 0.094^***^ (0.020)	− 0.018 (0.018)	− 0.039^*^ (0.022)	− 0.046^*^^*^ (0.022)				
Mix-DRG-4	− 0.117^***^ (0.022)	0.011 (0.025)	− 0.024 (0.033)	− 0.015 (0.033)				
Mix-DRG-6	− 0.001 (0.019)	0.127^***^ (0.023)	0.091^***^ (0.025)	0.089^***^ (0.025)				
Mix-FFS-8					− 0.050^***^ (0.016)	− 0.043^**^ (0.018)	− 0.070 (0.057)	− 0.079 (0.056)
Mix-FFS-6					− 0.184^***^ (0.025)	− 0.173^***^ (0.029)	− 0.217^**^ (0.098)	− 0.204^**^ (0.096)
$\left|s\right|$		0.038^***^ (0.004)	0.028^***^ (0.007)	0.028^***^ (0.007)		0.003 (0.005)	− 0.010 (0.028)	− 0.009 (0.027)
*B*			− 0.002 (0.016)	− 0.002 (0.015)			− 0.027 (0.019)	− 0.027 (0.018)
*C*			0.007 (0.015)	0.007 (0.015)			− 0.023 (0.019)	− 0.023 (0.018)
*y*			0.052^***^ (0.018)	0.052^***^ (0.017)			− 0.045 (0.056)	− 0.044 (0.055)
*z*			0.054^**^ (0.027)	0.054^**^ (0.026)			− 0.048 (0.107)	− 0.046 (0.105)
Intern experience				0.081^***^ (0.012)				0.072^***^ (0.016)
Female				0.053^***^ (0.014)				− 0.086^***^ (0.017)
Constant	0.794^***^ (0.007)	0.602^***^ (0.022)	0.619^***^ (0.030)	0.547^***^ (0.032)	0.870^***^ (0.007)	0.853^***^ (0.026)	0.967^***^ (0.191)	0.998^***^ (0.189)
Observations	1584	1584	1584	1584	984	984	984	984
*N*	88	88	88	88	55	55	55	55
*R*^2^	0.036	0.080	0.086	0.122	0.088	0.088	0.092	0.123

Robust standard errors are in parentheses

^***^p < 0.01

^**^p < 0.05

^*^p < 0.1

**Fig. 2:**
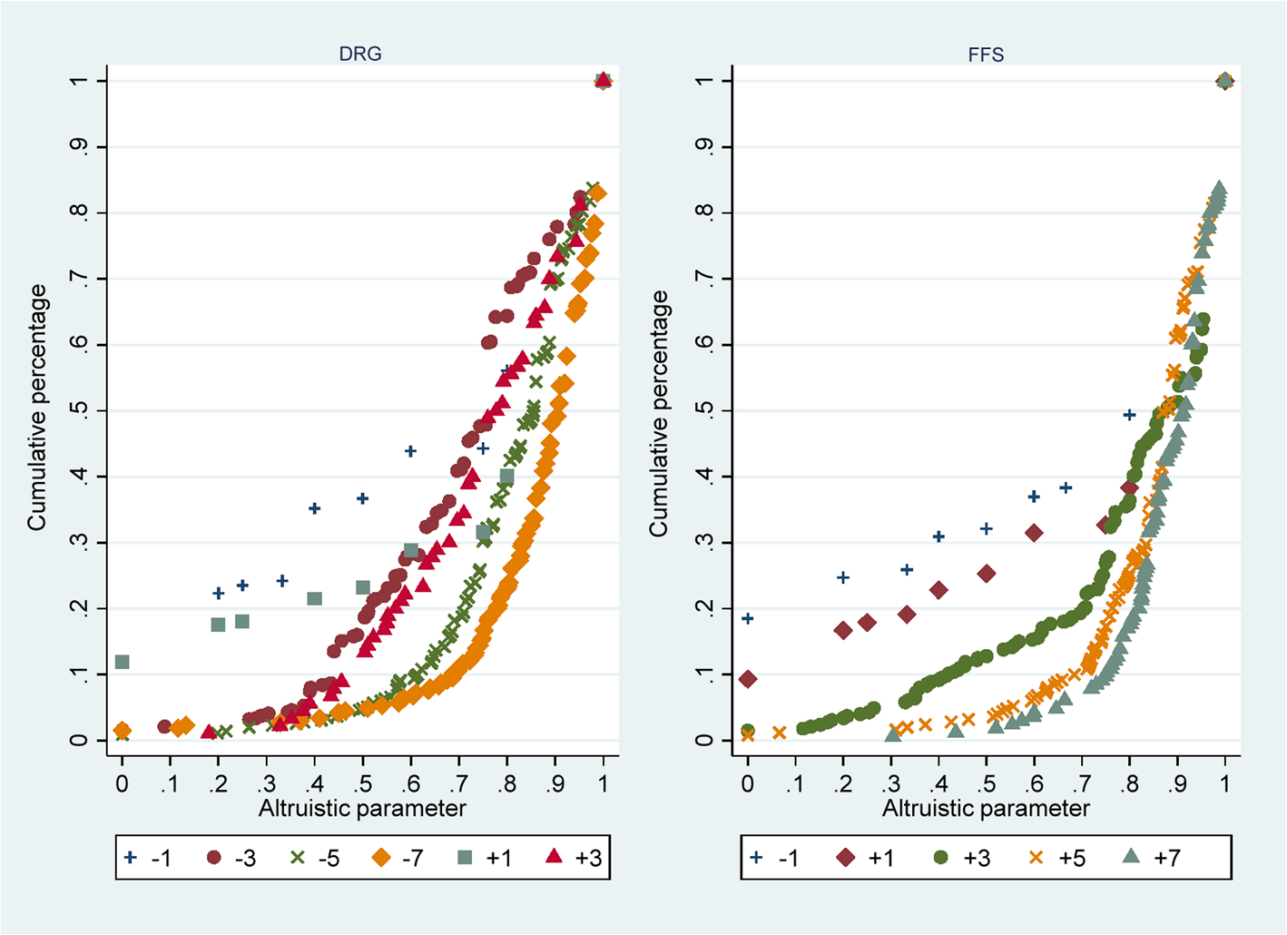
Cumulative frequency distribution graph of *α* under different trade-off ranges

The regression analysis of *α* showed that *α* was increased with the increase of the trade-off range in DRG payment systems. To further explain this effect, the cumulative frequency distribution graph of *α* under different relative trade-off ranges (*s *= q^ - q*) was plotted (see Fig. 2). As shown in the figure, a larger $\left|s\right|$ was, it was more conducive to the decrease of the proportion of physicians who fall below a certain level of *α*, which led to the improvement of the overall level of *α*. Under the same $\left|s\right|$, $+s$ was more effective than $-s$. The effect was more pronounced for low *α*’s but less obvious for high *α*’s because different points tended to overlap at high *α*.

### Physician altruism and patient health benefit

**Fig. 3:**
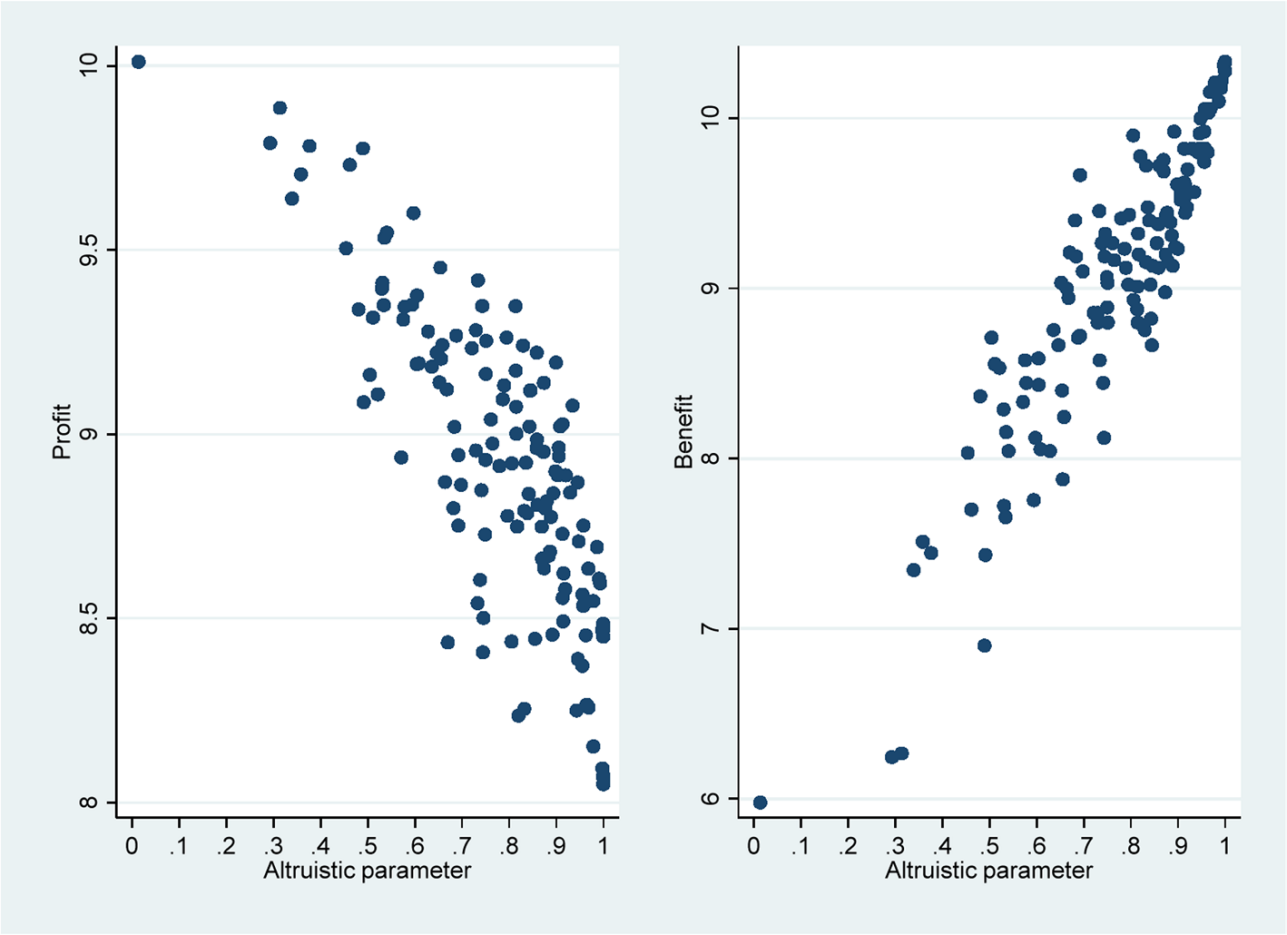
Scatter plot of the relationship between *α* and *π*(*q*) and *B*(*q*)

**Fig. 4:**
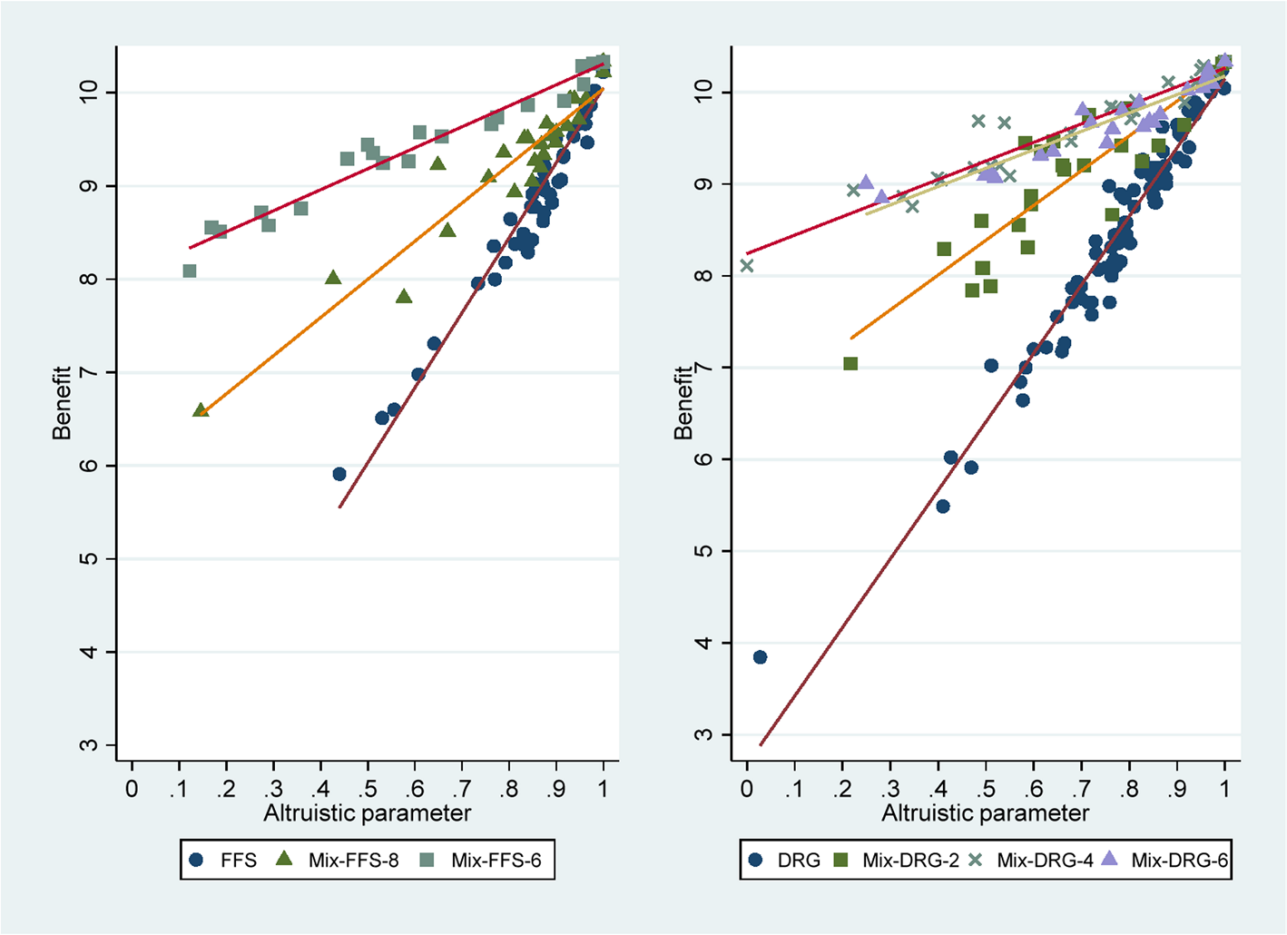
Scatter plot of the relationship between *α* and *B*(*q*) in the FFS and DRG payment systems

Considering the inconsistent direction of the improvement of physician behavior and the reduction of *α* after the transition from pure payment system to mixed payment scheme, we further analyzed the relationship between *α* and the behavioral indicators mentioned above. Taking *r* as an example, from the pure payment system to mixed payment scheme, the correlation coefficient with *α* increased from 0.839 to 0.925 (Spearman’s *ρ*, *p* < 0.01). The relationship between *α* and *π*(*q*) and *B*(*q*) is presented in a scatter plot (see Fig. 3). Of note, *α* had a significant negative correlation with *π*(*q*) (Spearman’s *ρ* =  − 0.777, *p* < 0.01) and a significant positive correlation with *B*(*q*) (Spearman’s *ρ* = 0.913, *p* < 0.01). Our previous study also showed an increase in *B*(*q*) when switching from the pure payment system to mixed payment scheme [39]. We further explored the relationship between *α* and *B*(*q*) in different payment systems. The scatter plot in Fig. 4 shows the differences more clearly. The horizontal axis of the Fig. 4 is *α*; the vertical axis is *B*(*q*); and different linear fitting lines represent different payment systems. According to the slope of different fitting lines is greater than zero, *B*(*q*) increases with the increase of *α*. In terms of the magnitude of the slope, the pure payment system was the largest, and the mixed payment scheme was relatively small. This implies that the same degree of change in *α* resulted in a smaller increase in *B*(*q*) in the mixed payment scheme than in the pure payment system. However, physicians with the same *α* generated more *B*(*q*) in the mixed payment scheme than in the pure payment system. If physicians generated equal *B*(*q*) in the mixed and pure payment system, then the *α* in the former was lower than that in the latter.

## Discussion

In this paper, we used a controlled laboratory experiment and a medical framework to explore the effects of exogenously changed payment system on physicians’ behavior and altruism. Based on the OLS regressions, we further implemented Tobit regressions to conduct robustness tests and yielded similar results (see additional file 3). In addition, the random effects model in our previous study also yielded similar results [39]. Therefore, we believe that causality is relatively reliable even if selection bias of subjects exists based on these analyses. Our results are consistent with previous experimental studies that mixed payment schemes reduce the underprovision in DRG payment system and the overprovision in FFS payment system [17, 43], and increase the ratio of optimal decisions, physicians’ profit, and patients’ benefit [39].

The two methods we used to quantify altruism can be complementary. Specifically, the *α* of 0.78 (s.d., 0.18) in our study was close to that of 0.75 (s.d., 0.26) in Brosig-Koch et al. [17]. The MRS of 1.078 was lower than 1.84 in Godager and Wiesen [26], mainly because *q*ˆ = 4 for *k* = 1 patient in their study was close to q^*^ = 5 under pure FFS payment system, while *q*ˆ = 10 for all types of patients in our study. This resulted in a reduced trade-off between q^*^ and *q*ˆ for physicians and a potential increase in MRS. The proportion of subjects with MRS greater than 1 (44.5%) was close to that of 44% in Godager and Wiesen [26]. In addition, there was a significant positive correlation between MRS and *α*. However, the estimate of MRS was easily limited by the estimated coefficient of *π*(*q*). When subjects always choose q^*^, the estimated coefficient of *π*(*q*) tends to 0 and the MRS cannot be estimated. The advantage is that the degree of altruism can be well distinguished. In contrast, *α* is calculable, except when out of the Pareto efficiency decision range. The disadvantage is that the degree of altruism is not defined. The two can be complementary, that is, when neither can be estimated, the other can be used to reflect the degree of altruism.

Intra-group comparison and regression analysis found that the *α* and MRS were lower under the mixed payment scheme than under the pure payment system, mainly because the design of the former was supposed to induce physician to provide quantity of medical service closer to q^*^ by reducing the trade-off between *q*ˆ and q^*^. However, the decrease of the trade-off range increased the proportion of physicians with lower *α*, which led to the decrease of the overall level of *α*. Taking the example of physician making decisions for *A*_*y*_ under pure DRG and Mix-DRG-4. Assuming that a physician with a lower (higher) *α* chooses to provide *q* = 1 (4) in pure DRG, the *α* is 0.33 (0.89). When in Mix-DRG-4, the same physician maybe chooses *q* = 4 (5), and then the *α* is 0 (1). In this case, except for the overall *α*, the deviation of medical service quantity, *B*(*q*) and *π*(*q*) are improved. More generally, when physicians choose the same quantity of medical service under the pure payment system and mixed payment scheme, the *α* is higher in the former than in the latter. Taking *A*_*z*_ as an example, if a physician chooses *q* = 5 under both pure DRG and Mix-DRG-4, then the *α* is 0.86 and 0.44 in the former and the latter, respectively. Similar finding is referred to as a devaluation of the weight on altruism in the study of Brosig-Koch et al. [44].

Whether in general or under different payment systems, *B*(*q*) increases with the increase of *α*. However, the increase in *B*(*q*) for each additional unit of *α* was smaller under the mixed payment scheme than under the pure payment system. Under the smaller trade-off range in the mixed payment scheme, the loss of the same unit of *π*(*q*) means a larger *α*. In other words, if the *α* is the same under the pure payment system and mixed payment scheme, it implies that physicians lose more units of *π*(*q*) under the former, and thereby increase more units of *B*(*q*). In the case of *A*_*z*_ in pure DRG and Mix-DRG-4, when the *α* increases from 0 to 0.76, physicians need to lose 4 units of *π*(*q*) in the former and 2 units in the latter, respectively. That is an increase of 4 units and 2 units of *B*(*q*). At the same *α*, the *B*(*q*) under the mixed payment scheme is still higher than that under the pure payment system because the *q*ˆ under the former is closer to the q^*^. Taking the above situation as an example, the q^*^ = 7, and the *q*ˆ = 0 and *q*ˆ = 4 under pure DRG and Mix-DRG-4, respectively. When the *α* is 0.76, physicians generate 4 units of *B*(*q*) in the former and 6 units in the latter.

The robustness test of the MRS on the *α* showed that the estimation of altruism is reliable. Previous studies have suggested that physicians can be paid based on altruism [26, 45]. Several theoretical studies argue that physicians who are more altruistic should be paid on CAP [9, 46], regardless of the severity of illness. Barham and Milliken [47] have shown that altruistic physicians who treat primarily frail patients should be paid on FFS, whereas nonaltruistic physicians who treat healthy patients should be paid on CAP. Several experimental studies have shown that physicians’ altruistic behavior is affected by the severity of illness or medical needs [17, 48]. Liu and Ma [12] showed that a type of physician could be revealed by selecting an item from the full cost-share-transfer menu, and a more altruistic physician should receive a larger lump-sum transfer ex ante.

Our study revealed that physicians’ altruism is influenced by the trade-off range. A larger and a positive trade-off range are conducive to decreased proportion of physicians who fall below a certain level of altruism. Therefore, a payment system with a positive larger trade-off range can be designed for physicians with a lower degree of altruism to enhance the overall level of altruism and ensure *B*(*q*). However, physicians with a higher degree of altruism are less affected by the trade-off range, we can design a payment system with a smaller trade-off range to maintain their higher level of altruism without causing *π*(*q*) to decrease too much due to the expansion of the trade-off range.

In view of the above theoretical inspirations of the design of payment system and the improved physician behavior and reduced altruistic parameters under the mixed payment schemes, we have reason to believe that payment systems that align physician interests with patient benefits will help increase both while maintaining physician altruism. Therefore, if appropriate economic and non-economic incentives are given to physicians’ pro-patient behaviors under the existing payment systems, it will help to realize the unity of the interests of physicians and patients, and thus contribute to the realization of patient-centered value medicine.

## Conclusion

Our study proves that the mixed payment schemes improve physicians’ behavior and relate to lower altruistic parameter. The decrease of altruistic parameter can be explained by the decrease of trade-off range and the devaluation of the weight on altruism under the mixed payment schemes. In future studies, we intend to realize the alignment of interests between physicians and patients by designing the same service quantity for maximizing physicians’ profit and patients’ benefit, and simulate economic and non-economic incentives through pay-for-performance and public report on the behavior of physicians in providing the optimal service quantity for patients. On this basis, laboratory intervention experiments were conducted to test the effect of the above design on physicians’ behavior and altruism, so as to provide reference for the reform of payment systems.

### Limitations

On the basis of reliable measurement of physician altruism, this study reveals the change of altruism when switching from the pure payment system to mixed payment scheme and analyzes the reasons for the change. However, there are also some limitations in our study. First, we did not specify the criteria for the accuracy of the revealed types of altruistic physicians; second, we did not define the optimal range of trade-off for physicians with different levels of altruism; and finally, whether this range is stable enough or will be affected by the severity of illness was not explored. In addition, our study was based on a narrow set of parameters; thus, applying the experimental results to the design of real-world physicians’ payment systems is limited.

## Supplementary Information

**Additional file 1. **Instructionsfot the experiment.

**Additional file 2: Fig. S1. **Decision screen shotfor patient B_y_ in DRG. **Fig. S2.** Decision screen shotfor patient B_y_ in Mix-DRG-2. **Fig. S3.** Decision screen shotfor patient B_y_ in FFS. **Fig. S4.** Decision screen shotfor patient B_y_ in Mix-FFS-8.

**Additional file 3: Table S1. **Unstandardized *α* in DRG payment system. **Table S2.** Unstandardized *α* in FFS payment system. **Table S3.** Tobit regression of |d|. **Table S4.** Tobit regression of α. 

## Data Availability

The data of this study are available from the corresponding author, YLH, upon reasonable request.

## Abbreviations

DRG: Diagnosis-related groups
P4P: Pay for performance
DG: Dictator game
FFS: Fee-for-service
CAP: Capitation
SAL: Salary
MRS: Marginal rate of substitution
WSR: Wilcoxon signed-rank
MWU: Mann–Whitney U
KWH: Kruskal–Wallis H
OLS: Ordinary least squares
