# Supplementary material for: Physician altruism under the change from pure payment system to mixed payment schemes: experimental evidence
Source: BMC Health Serv Res. 2023 Feb 2;23:111. doi: 10.1186/s12913-023-09112-4 (PMC9893586; doi:10.1186/s12913-023-09112-4)
Supplement: Supplementary file 1 — Additional file 1. Instructionsfot the experiment. [file 12913_2023_9112_MOESM1_ESM.doc]

**Instructions for the experiment**

**Preface**

Thank you for participating in this payment economics experiment. In the following tasks, you need to make a series of decisions, which will determine your earnings. It is therefore very important that you read the instructions carefully. Your decisions will be made anonymous based on the computer interface. During the experiment, you are not allowed to communicate with each other. If you have any questions, please raise your hand and an experimenter will answer them privately for you. The currencies in the experiment are stated in Taler. Your aggregate earnings depend on all decisions and will be paid to you according to the ratio of 10 Taler to 1 RMB. If you successfully complete the experiment, you will get a basic reward of 30 RMB. The experiment consists of two parts (Part I and Part II) and lasts about 90 minutes. Part I is a pure payment system comprising diagnosis-related groups (DRG) or fee-for-service (FFS). Part II is a mixed payment scheme consisting of DRG and FFS. Note that none of your decisions in either part have any influence on the other part.

**Your experimental decision-making**

In each part of the experiment, you play a physician to make a decision on your computer screen, where the quantity of medical services provided is an integer between 0 and 10, for nine different patients. The scenario is designed to comprehensively consider three illnesses, *A*, *B*, and *C*, and three illness severities, *x*, *y*, and *z*. The experiment is conducted in five rounds with 18 decisions in each round (nine in Part I and nine in Part II), and you will make a total of 90 decisions.

You need to input the quantity of medical services you want to provide in the box of “Please indicate the quantity of medical services you want to provide” and then click the “OK” button in the lower right corner of the screen. When you make a decision, the computer screen will display the detailed information, including your remuneration, cost, your profit, and patient benefit, corresponding to the quantity of medical services. In the DRG payment system, you get a different lump sum for each patient (*A_x_*…*C_z_*), and in the FFS payment system, you get a different fee for each service for each illness (*A*, *B*, and *C*). The costs increase as the quantity of medical services increases. Your profit is equal to remuneration minus cost.

**Payment**

At the end of the experiment, you will be paid the sum of the profit from all your 90 decisions. The benefit of virtual patients generated by you is randomly selected in one round from the five rounds to be donated to the Red Cross Society of China. To ensure the authenticity of the donation, we will randomly select one participant to supervise the whole donation process after the end of the experiment, and the supervisor will get an additional 50 RMB.

**Next, you need to answer some comprehension questions and participate in a pilot experiment that will familiarize you with the experimental situation!**

**Comprehension questions**

To help you better understand the decision situation of the experiment, we set up a series of comprehension questions. After these questions are answered correctly, the pilot experiment can be conducted. When you have completed the pilot experiment, the formal experiment will be conducted. In the formal experiment, only when all of you have completed the decision for the same patient, you can move to the decision for the next patient.

**Screen 1:** The upper left of the screen shows the payment system and patient type, such as “Payment system: diagnosis-related groups (DRG); Patient: illness *A*; Severity: *y*”. The middle of the screen consists of the information about “Your payment”, “Your cost”, “Your profit”, and “Patient’s benefit”, depending on the different quantity of medical services that you will provide.

The questions you need to answer are as follows:

Suppose you provide that the quantity of medical services is 4. Please answer the following questions:

(i) How much is “Your payment”?

(ii) How much is “Your cost”?

(iii) How much is “Your profit”?

(iii) How much is “Patient’s benefit”?

Please fill the answers to the above questions in the four corresponding boxes at the bottom of the screen. If the answer is correct, click the “OK” button in the lower right corner of the screen; you will automatically enter screen 2. If your answer is wrong, there will be a prompt. Please continue to read the information on the screen carefully and answer again until your answer is correct, and then you can enter screen 2.

**Screen 2:** The upper left of the screen shows the payment system and patient type, such as “Payment system: diagnosis-related groups (DRG); Patient: illness *A*; Severity: *y*”. The middle of the screen consists of the information about “Your payment”, “Your cost”, “Your profit”, “Patient’s benefit” depending on the different quantity of medical services that you will provide.

The questions you need to answer are as follows:

(i) What is the quantity of medical services you need to provide when “Your profit” is at maximum?__________

(ii) What is the quantity of medical services you need to provide when “Patient’s benefit” is at maximum?__________

Please fill the answers to the above questions in the two corresponding boxes at the bottom of the screen. If the answer is correct, click the “OK” button in the lower right corner of the computer screen, and you will automatically enter the pilot experiment. If your answer is wrong, there will be a prompt. Please continue reading the information on the screen carefully and answer again until you enter the pilot experiment.

**Instructions for the pilot experiment**

The pilot experiment is the simulation of the formal experiment.

The upper left of the screen shows the payment system and patient type, such as “Payment system: diagnosis-related groups (DRG); Patient: illness *A*; Severity: *y*”. The middle of the screen consists of the information about “Your payment”, “Your cost”, “Your profit”, and “Patient’s benefit”, depending on the quantity of medical services that you will provide. Based on this information, you need to decide the quantity of medical services to be provided for each patient. When you are making a decision, a prompt will appear at the bottom of the screen, such as “Facing with patient *A_y_*, the quantity of medical services you want to provide is”. Please enter an integer from 0 to 10 to represent the number of medical services you wish to provide in the box behind the prompt.

After typing the quantity of medical services, click the “OK” button in the lower right corner of the screen; you will automatically enter the results output screen. At this moment, two lines appear on the screen, the first line is “Your profit is XX”, and the second line is “Patient’s benefit is XX”. In the formal experiment, you need to fill these data into the corresponding cell in the experiment record sheet. After filling them, click the “OK” button in the lower right corner of the screen, and you will automatically move on to the next decision.

During the pilot experiment, if you have any questions, please raise your hand, and an experimenter will answer them privately for you. The formal experiment will start after the pilot experiment has been completed.

Finally, to prevent other participants after you from being affected by your choices, please do not disclose the content of this experiment to others. Thank you for your collaboration!
