# Supplementary material for: Physician altruism under the change from pure payment system to mixed payment schemes: experimental evidence
Source: BMC Health Serv Res. 2023 Feb 2;23:111. doi: 10.1186/s12913-023-09112-4 (PMC9893586; doi:10.1186/s12913-023-09112-4)
Supplement: Supplementary file 2 — Additional file 2: Fig. S1. Decision screen shotfor patient By in DRG. Fig. S2. Decision screen shotfor patient By in Mix-DRG-2. Fig. S3. Decision screen shotfor patient By in FFS. Fig. S4. Decision screen shotfor patient By in Mix-FFS-8. [file 12913_2023_9112_MOESM2_ESM.docx]

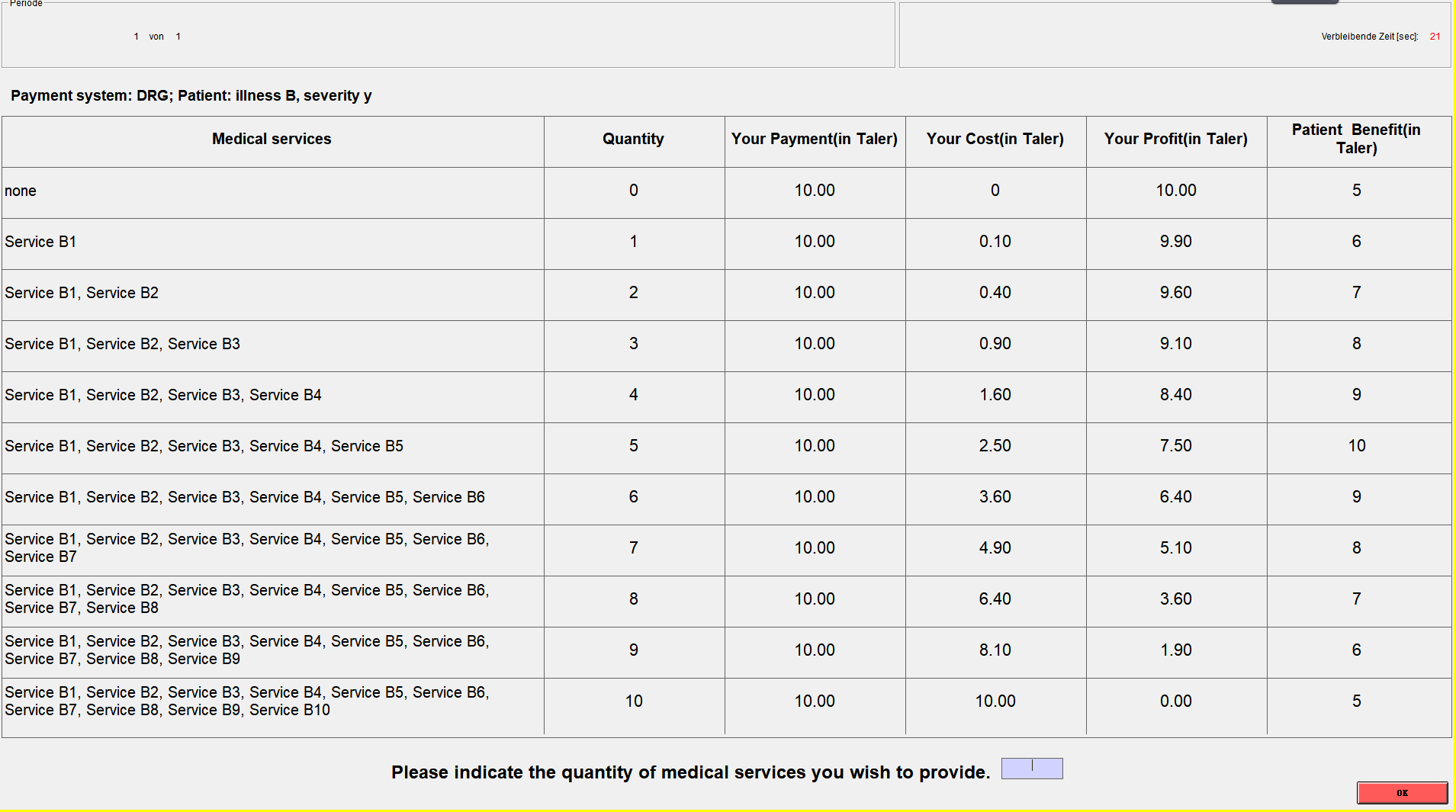


**Fig. S1.** Decision screen shot for patient *B_y_* in DRG


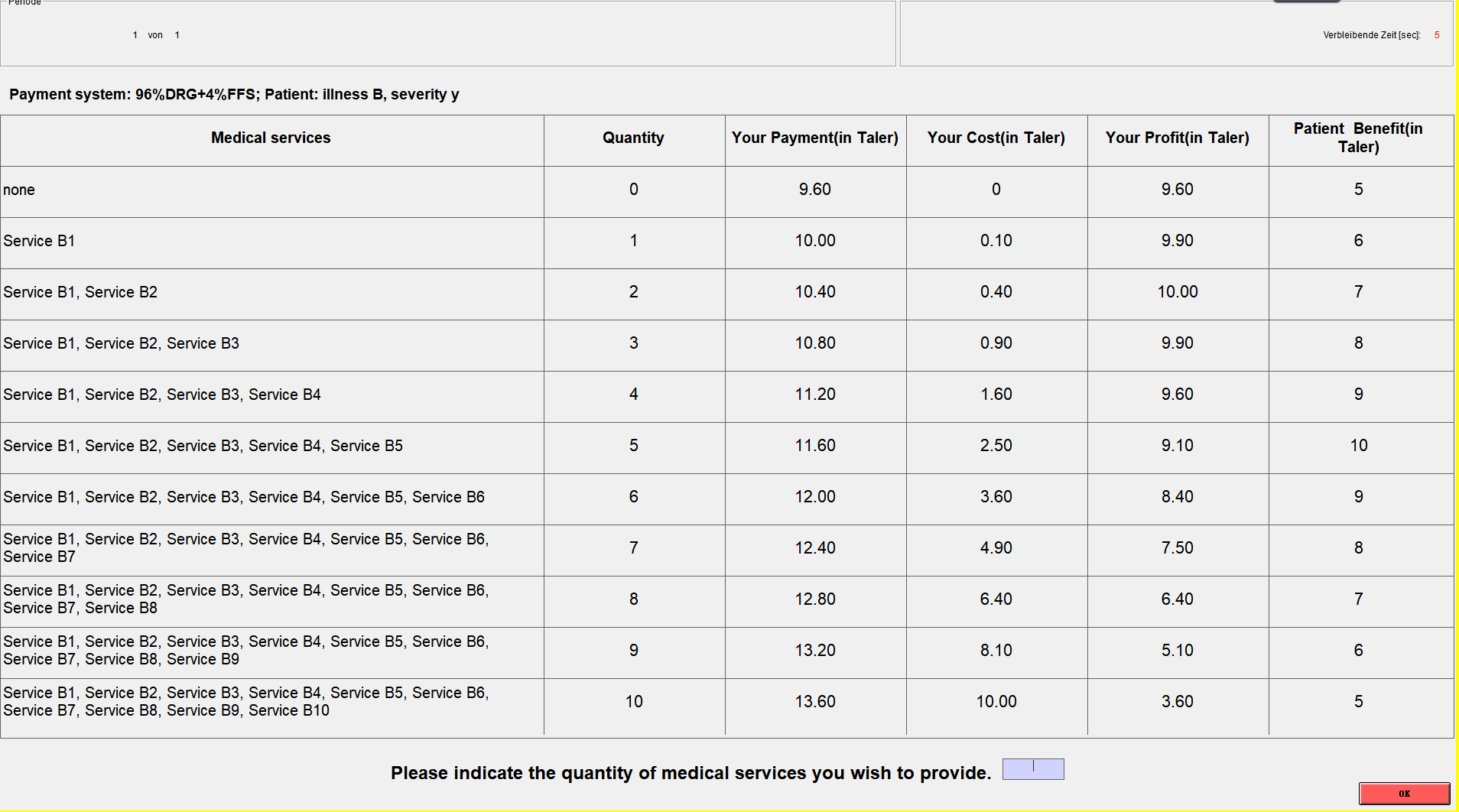


**Fig. S2.** Decision screen shot for patient *B_y_* in Mix-DRG-2


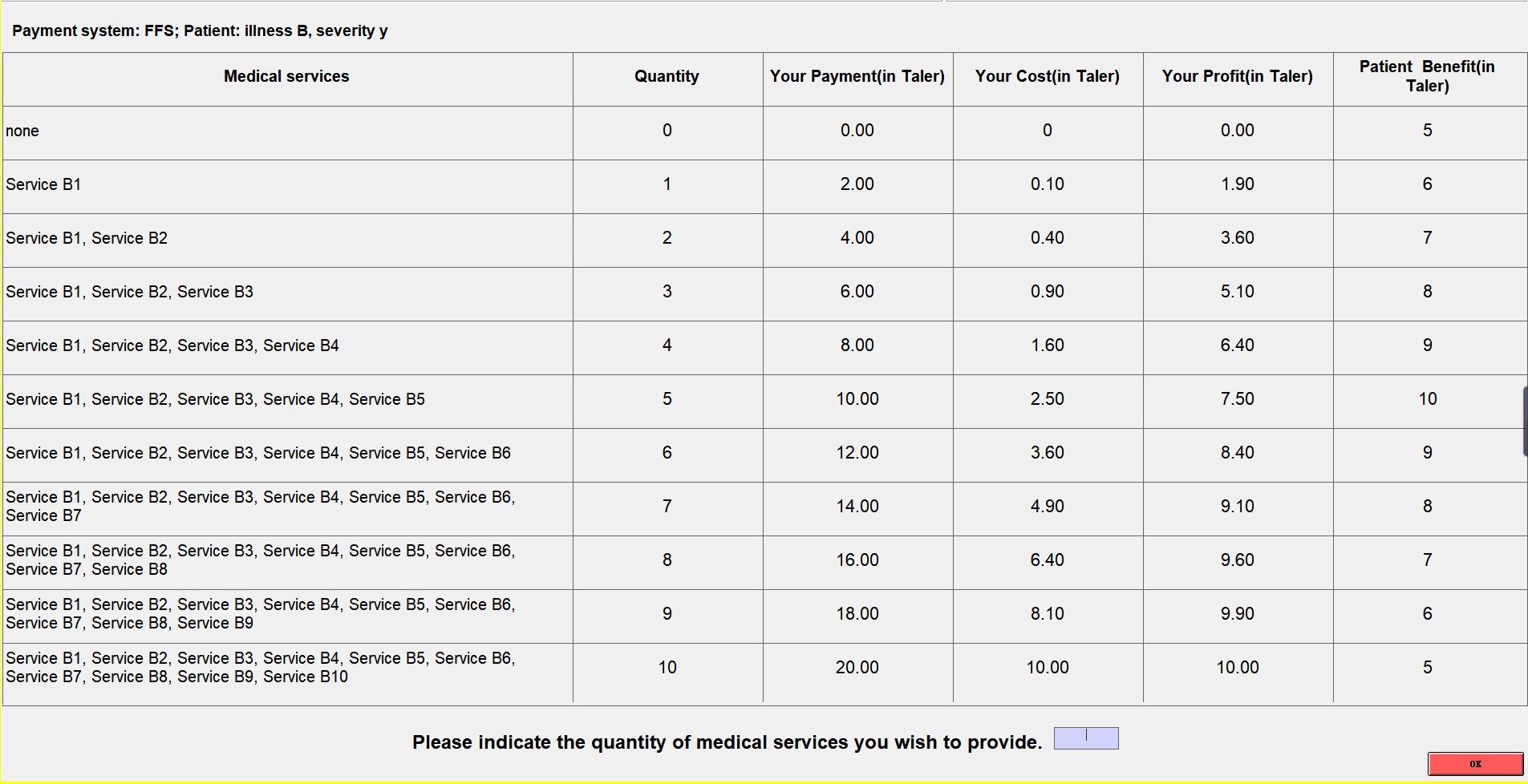


**Fig. S3.** Decision screen shot for patient *B_y_* in FFS


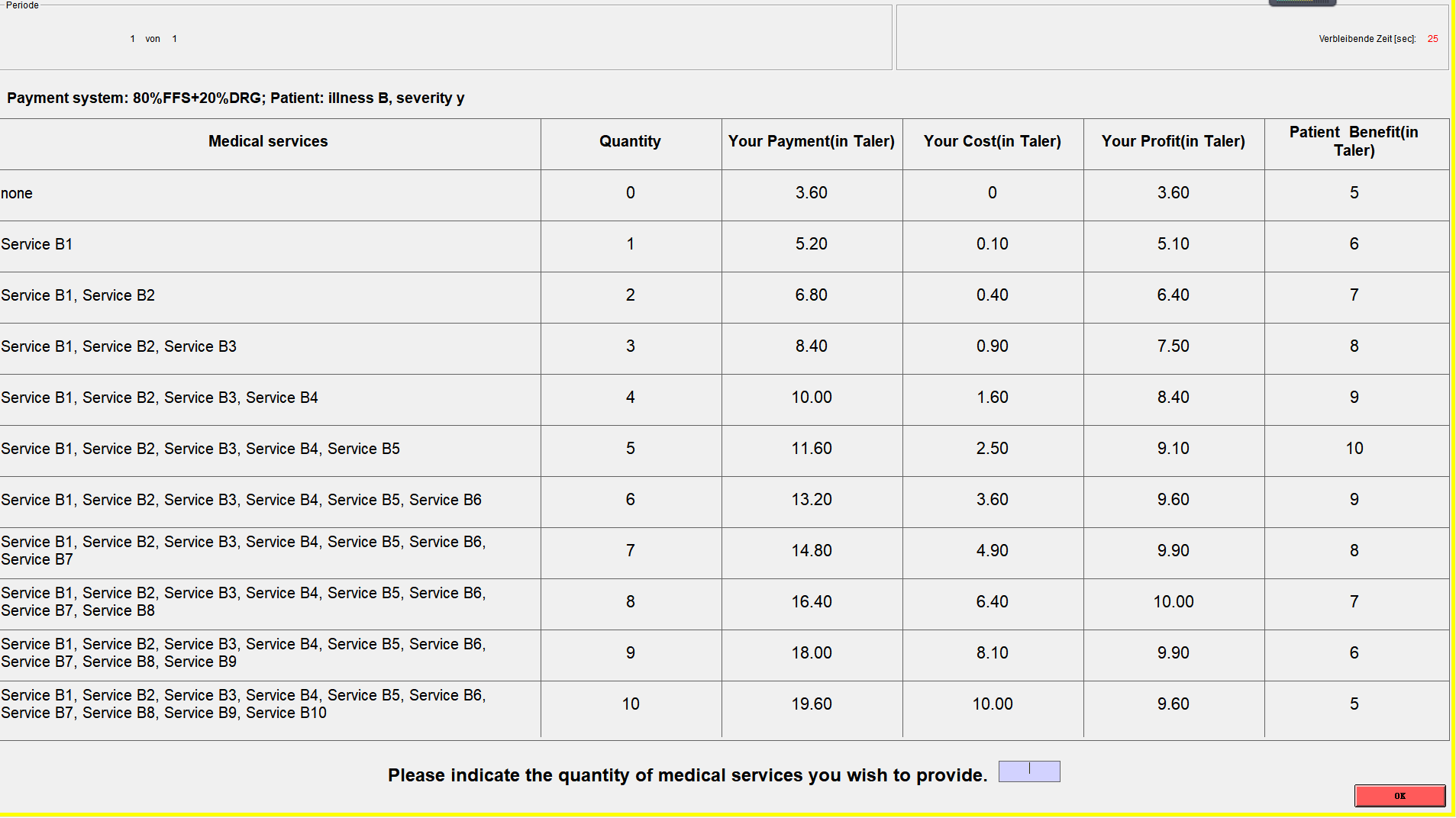


**Fig. S4.** Decision screen shot for patient *B_y_* in Mix-FFS-8
