# Supplementary material for: Physician altruism under the change from pure payment system to mixed payment schemes: experimental evidence
Source: BMC Health Serv Res. 2023 Feb 2;23:111. doi: 10.1186/s12913-023-09112-4 (PMC9893586; doi:10.1186/s12913-023-09112-4)
Supplement: Supplementary file 3 — Additional file 3: Table S1. Unstandardized α in DRG payment system. Table S2. Unstandardized α in FFS payment system. Table S3. Tobit regression of |d|. Table S4. Tobit regression of α. [file 12913_2023_9112_MOESM3_ESM.docx]

**Table S1** Unstandardized *α* in DRG payment system

| Payment system | Patient | *θ* | 1 − *μ* | *p* | *q* ≤ *q*^*^ | *q* ≥ *q*^*^ |
| --- | --- | --- | --- | --- | --- | --- |
| DRG | *A_x_* | 1 | 0 | ‒ | 0.2*q*/(0.2*q* + 1) | 0.2*q*/(0.2*q* − 1) |
|  | *A_y_* | 1 | 0 | ‒ | 0.2*q*/(0.2*q* + 1) | 0.2*q*/(0.2*q* − 1) |
|  | *A_z_* | 1 | 0 | ‒ | 0.2*q*/(0.2*q* + 1) | 0.2*q*/(0.2*q* − 1) |
|  | *B_x_* | 1 | 0 | ‒ | 0.2*q*/(0.2*q* + 1) | 0.2*q*/(0.2*q* − 1) |
|  | *B_y_* | 1 | 0 | ‒ | 0.2*q*/(0.2*q* + 1) | 0.2*q*/(0.2*q* − 1) |
|  | *B_z_* | 1 | 0 | ‒ | 0.2*q*/(0.2*q* + 1) | 0.2*q*/(0.2*q* − 1) |
|  | *C_x_* | 2 | 0 | ‒ | 0.2*q*/(0.2*q* + 2) | 0.2*q*/(0.2*q* − 2) |
|  | *C_y_* | 2 | 0 | ‒ | 0.2*q*/(0.2*q* + 2) | 0.2*q*/(0.2*q* − 2) |
|  | *C_z_* | 2 | 0 | ‒ | 0.2*q*/(0.2*q* + 2) | 0.2*q*/(0.2*q* − 2) |
| Mix-DRG-2 | *A_x_* | 1 | 0.03 | 13.33 | (0.2*q* − 0.4)/(0.2*q* + 0.6) | (0.2*q* − 0.4)/(0.2*q* − 1.4) |
|  | *A_y_* | 1 | 0.04 | 10.00 | (0.2*q* − 0.4)/(0.2*q* + 0.6) | (0.2*q* − 0.4)/(0.2*q* − 1.4) |
|  | *A_z_* | 1 | 0.05 | 8.00 | (0.2*q* − 0.4)/(0.2*q* + 0.6) | (0.2*q* − 0.4)/(0.2*q* − 1.4) |
|  | *B_x_* | 1 | 0.03 | 13.33 | (0.2*q* − 0.4)/(0.2*q* + 0.6) | (0.2*q* − 0.4)/(0.2*q* − 1.4) |
|  | *B_y_* | 1 | 0.04 | 10.00 | (0.2*q* − 0.4)/(0.2*q* + 0.6) | (0.2*q* − 0.4)/(0.2*q* − 1.4) |
|  | *B_z_* | 1 | 0.05 | 8.00 | (0.2*q* − 0.4)/(0.2*q* + 0.6) | (0.2*q* − 0.4)/(0.2*q* − 1.4) |
|  | *C_x_* | 2 | 0.03 | 13.33 | (0.2*q* − 0.4)/(0.2*q* + 1.6) | (0.2*q* − 0.4)/(0.2*q* − 2.4) |
|  | *C_y_* | 2 | 0.04 | 10.00 | (0.2*q* − 0.4)/(0.2*q* + 1.6) | (0.2*q* − 0.4)/(0.2*q* − 2.4) |
|  | *C_z_* | 2 | 0.05 | 8.00 | (0.2*q* − 0.4)/(0.2*q* + 1.6) | (0.2*q* − 0.4)/(0.2*q* − 2.4) |
| Mix-DRG-4 | *A_x_* | 1 | 0.05 | 5.33 | (0.2*q* − 0.27)/(0.2*q* + 0.73) | (0.2*q* − 0.27)/(0.2*q* − 1.27) |
|  | *A_y_* | 1 | 0.06 | 5.00 | (0.2*q* − 0.30)/(0.2*q* + 0.70) | (0.2*q* − 0.30)/(0.2*q* − 1.30) |
|  | *A_z_* | 1 | 0.07 | 4.71 | (0.2*q* − 0.33)/(0.2*q* + 0.67) | (0.2*q* − 0.33)/(0.2*q* − 1.33) |
|  | *B_x_* | 1 | 0.05 | 5.33 | (0.2*q* − 0.27)/(0.2*q* + 0.73) | (0.2*q* − 0.27)/(0.2*q* − 1.27) |
|  | *B_y_* | 1 | 0.06 | 5.00 | (0.2*q* − 0.30)/(0.2*q* + 0.70) | (0.2*q* − 0.30)/(0.2*q* − 1.30) |
|  | *B_z_* | 1 | 0.07 | 4.71 | (0.2*q* − 0.33)/(0.2*q* + 0.67) | (0.2*q* − 0.33)/(0.2*q* − 1.33) |
|  | *C_x_* | 2 | 0.05 | 5.33 | (0.2*q* − 0.27)/(0.2*q* + 1.73) | (0.2*q* − 0.27)/(0.2*q* − 2.27) |
|  | *C_y_* | 2 | 0.06 | 5.00 | (0.2*q* − 0.30)/(0.2*q* + 1.70) | (0.2*q* − 0.30)/(0.2*q* − 2.30) |
|  | *C_z_* | 2 | 0.07 | 4.71 | (0.2*q* − 0.33)/(0.2*q* + 1.67) | (0.2*q* − 0.33)/(0.2*q* − 2.33) |
| Mix-DRG-6 | *A_x_* | 1 | 0.35 | 3.43 | (0.2*q* − 1.2)/(0.2*q* − 0.2) | (0.2*q* − 1.2)/(0.2*q* − 2.2) |
|  | *A_y_* | 1 | 0.36 | 3.33 | (0.2*q* − 1.2)/(0.2*q* − 0.2) | (0.2*q* − 1.2)/(0.2*q* − 2.2) |
|  | *A_z_* | 1 | 0.37 | 3.24 | (0.2*q* − 1.2)/(0.2*q* − 0.2) | (0.2*q* − 1.2)/(0.2*q* − 2.2) |
|  | *B_x_* | 1 | 0.35 | 3.43 | (0.2*q* − 1.2)/(0.2*q* − 0.2) | (0.2*q* − 1.2)/(0.2*q* − 2.2) |
|  | *B_y_* | 1 | 0.36 | 3.33 | (0.2*q* − 1.2)/(0.2*q* − 0.2) | (0.2*q* − 1.2)/(0.2*q* − 2.2) |
|  | *B_z_* | 1 | 0.37 | 3.24 | (0.2*q* − 1.2)/(0.2*q* − 0.2) | (0.2*q* − 1.2)/(0.2*q* − 2.2) |
|  | *C_x_* | 2 | 0.35 | 3.43 | (0.2*q* − 1.2)/(0.2*q* + 0.8) | (0.2*q* − 1.2)/(0.2*q* − 3.2) |
|  | *C_y_* | 2 | 0.36 | 3.33 | (0.2*q* − 1.2)/(0.2*q* + 0.8) | (0.2*q* − 1.2)/(0.2*q* − 3.2) |
|  | *C_z_* | 2 | 0.37 | 3.24 | (0.2*q* − 1.2)/(0.2*q* + 0.8) | (0.2*q* − 1.2)/(0.2*q* − 3.2) |

**Table S2** Unstandardized *α* in FFS payment system

| Payment system | Patient | *θ* | 1 − *μ* | *p* | *q* ≤ *q*^*^ | *q* ≥ *q*^*^ |
| --- | --- | --- | --- | --- | --- | --- |
| FFS | *A_x_* | 1 | 1 | 1.91 | (0.2*q* − 1.91)/(0.2*q* − 0.91) | (0.2*q* − 1.91)/(0.2*q* − 2.91) |
|  | *A_y_* | 1 | 1 | 1.91 | (0.2*q* − 1.91)/(0.2*q* − 0.91) | (0.2*q* − 1.91)/(0.2*q* − 2.91) |
|  | *A_z_* | 1 | 1 | 1.91 | (0.2*q* − 1.91)/(0.2*q* − 0.91) | (0.2*q* − 1.91)/(0.2*q* − 2.91) |
|  | *B_x_* | 1 | 1 | 2 | (0.2*q* − 2)/(0.2*q* − 1) | (0.2*q* − 2)/(0.2*q* − 3) |
|  | *B_y_* | 1 | 1 | 2 | (0.2*q* − 2)/(0.2*q* − 1) | (0.2*q* − 2)/(0.2*q* − 3) |
|  | *B_z_* | 1 | 1 | 2 | (0.2*q* − 2)/(0.2*q* − 1) | (0.2*q* − 2)/(0.2*q* − 3) |
|  | *C_x_* | 2 | 1 | 2.1 | (0.2*q* − 2.1)/(0.2*q* − 0.1) | (0.2*q* − 2)/(0.2*q* − 4.1) |
|  | *C_y_* | 2 | 1 | 2.1 | (0.2*q* − 2.1)/(0.2*q* − 0.1) | (0.2*q* − 2)/(0.2*q* − 4.1) |
|  | *C_z_* | 2 | 1 | 2.1 | (0.2*q* − 2.1)/(0.2*q* − 0.1) | (0.2*q* − 2)/(0.2*q* − 4.1) |
| Mix-FFS-8 | *A_x_* | 1 | 0.79 | 1.91 | (0.2*q* − 1.51)/(0.2*q* − 0.51) | (0.2*q* − 1.51)/(0.2*q* − 2.51) |
|  | *A_y_* | 1 | 0.80 | 1.91 | (0.2*q* − 1.53)/(0.2*q* − 0.53) | (0.2*q* − 1.53)/(0.2*q* − 2.53) |
|  | *A_z_* | 1 | 0.81 | 1.91 | (0.2*q* − 1.55)/(0.2*q* − 0.55) | (0.2*q* − 1.55)/(0.2*q* − 2.55) |
|  | *B_x_* | 1 | 0.79 | 2.00 | (0.2*q* − 1.58)/(0.2*q* − 0.58) | (0.2*q* − 1.58)/(0.2*q* − 2.58) |
|  | *B_y_* | 1 | 0.80 | 2.00 | (0.2*q* − 1.60)/(0.2*q* − 0.60) | (0.2*q* − 1.60)/(0.2*q* − 2.60) |
|  | *B_z_* | 1 | 0.81 | 2.00 | (0.2*q* − 1.62)/(0.2*q* − 0.62) | (0.2*q* − 1.62)/(0.2*q* − 2.62) |
|  | *C_x_* | 2 | 0.79 | 2.10 | (0.2*q* − 1.66)/(0.2*q* + 0.34) | (0.2*q* − 1.66)/(0.2*q* − 3.66) |
|  | *C_y_* | 2 | 0.80 | 2.10 | (0.2*q* − 1.68)/(0.2*q* + 0.32) | (0.2*q* − 1.68)/(0.2*q* − 3.68) |
|  | *C_z_* | 2 | 0.81 | 2.10 | (0.2*q* − 1.70)/(0.2*q* + 0.30) | (0.2*q* − 1.70)/(0.2*q* − 3.70) |
| Mix-FFS-6 | *A_x_* | 1 | 0.59 | 1.91 | (0.2*q* − 1.13)/(0.2*q* − 0.13) | (0.2*q* − 1.13)/(0.2*q* − 2.13) |
|  | *A_y_* | 1 | 0.60 | 1.91 | (0.2*q* − 1.15)/(0.2*q* − 0.15) | (0.2*q* − 1.15)/(0.2*q* − 2.15) |
|  | *A_z_* | 1 | 0.61 | 1.91 | (0.2*q* − 1.17)/(0.2*q* − 0.17) | (0.2*q* − 1.17)/(0.2*q* − 2.17) |
|  | *B_x_* | 1 | 0.59 | 2.00 | (0.2*q* − 1.18)/(0.2*q* − 0.18) | (0.2*q* − 1.18)/(0.2*q* − 2.18) |
|  | *B_y_* | 1 | 0.60 | 2.00 | (0.2*q* − 1.20)/(0.2*q* − 0.20) | (0.2*q* − 1.20)/(0.2*q* − 2.20) |
|  | *B_z_* | 1 | 0.61 | 2.00 | (0.2*q* − 1.22)/(0.2*q* − 0.22) | (0.2*q* − 1.22)/(0.2*q* − 2.22) |
|  | *C_x_* | 2 | 0.59 | 2.10 | (0.2*q* − 1.24)/(0.2*q* + 0.76) | (0.2*q* − 1.24)/(0.2*q* − 3.24) |
|  | *C_y_* | 2 | 0.60 | 2.10 | (0.2*q* − 1.26)/(0.2*q* + 0.74) | (0.2*q* − 1.26)/(0.2*q* − 3.26) |
|  | *C_z_* | 2 | 0.61 | 2.10 | (0.2*q* − 1.28)/(0.2*q* + 0.72) | (0.2*q* − 1.28)/(0.2*q* − 3.28) |

**Table S3** Tobit regression of $\left| d \right|$

| Variable | 1. DRG | | |  | 1. FFS | | |
| --- | --- | --- | --- | --- | --- | --- | --- |
|  | 1 | 2 | 3 |  | 4 | 5 | 6 |
| Mix-DRG-2 | −0.493^***^  (0.082) | −0.493^***^  (0.080) | −0.464^***^  (0.079) |  |  |  |  |
| Mix-DRG-4 | −0.880^***^  (0.077) | −0.883^***^  (0.074) | −0.923^***^  (0.075) |  |  |  |  |
| Mix-DRG-6 | −1.278^***^  (0.088) | −1.262^***^  (0.089) | −1.252^***^  (0.086) |  |  |  |  |
| Mix-FFS-8 |  |  |  |  | −0.284^***^  (0.093) | −0.297^***^  (0.084) | −0.278^***^  (0.084) |
| Mix-FFS-6 |  |  |  |  | −0.524^***^  (0.095) | −0.505^***^  (0.091) | −0.526^***^  (0.092) |
| *B* |  | 0.035  (0.070) | 0.034  (0.069) |  |  | 0.233^***^  (0.085) | 0.234^***^  (0.085) |
| *C* |  | −0.124^*^  (0.071) | −0.125^*^  (0.069) |  |  | 0.198^**^  (0.085) | 0.198^**^  (0.084) |
| *y* (intermediate) |  | 0.097  (0.060) | 0.096  (0.059) |  |  | −0.480^***^  (0.088) | −0.481^***^  (0.088) |
| *z* (severe) |  | 0.354^***^  (0.074) | 0.353^***^  (0.073) |  |  | −1.000^***^  (0.093) | −1.004^***^  (0.093) |
| Intern experience |  |  | −0.350^***^  (0.059) |  |  |  | −0.118  (0.072) |
| Female |  |  | −0.270^***^  (0.066) |  |  |  | 0.253^***^  (0.078) |
| Constant | 1.215^***^  (0.042) | 1.096^***^  (0.061) | 1.432^***^  (0.077) |  | 0.789^***^  (0.052) | 1.146^***^  (0.086) | 1.008^***^  (0.099) |
| Observations | 1620 | 1620 | 1620 |  | 1080 | 1080 | 1080 |
| *N* | 90 | 90 | 90 |  | 60 | 60 | 60 |
| Pseudo *R*^2^ | 0.055 | 0.062 | 0.075 |  | 0.011 | 0.055 | 0.059 |

Robust standard errors are in parentheses. ^***^*p* < 0.01, ^**^*p* < 0.05, ^*^*p* < 0.1. The lower and the upper limits are the minimum and maximum of $\left| d \right|$, respectively.

**Table S4** Tobit regression of *α*

| Variable | A. DRG | | | |  | B. FFS | | | |
| --- | --- | --- | --- | --- | --- | --- | --- | --- | --- |
|  | 1 | 2 | 3 | 4 |  | 5 | 6 | 7 | 8 |
| Mix-DRG-2 | −0.081^***^  (0.026) | −0.010  (0.025) | −0.061^*^  (0.032) | −0.071^**^  (0.031) |  |  |  |  |  |
| Mix-DRG-4 | −0.094^***^  (0.031) | 0.024  (0.034) | −0.061  (0.050) | −0.050  (0.049) |  |  |  |  |  |
| Mix-DRG-6 | 0.087^***^  (0.031) | 0.201^***^  (0.034) | 0.123^***^  (0.035) | 0.119^***^  (0.035) |  |  |  |  |  |
| Mix-FFS-8 |  |  |  |  |  | −0.036  (0.025) | −0.077^***^  (0.028) | −0.158^*^  (0.088) | −0.171^**^  (0.087) |
| Mix-FFS-6 |  |  |  |  |  | −0.170^***^  (0.038) | −0.241^***^  (0.046) | −0.380^**^  (0.154) | −0.362^**^  (0.151) |
| $\left\vert s \right\vert$ |  | 0.036^***^  (0.006) | 0.011  (0.011) | 0.011  (0.011) |  |  | −0.021^***^  (0.007) | −0.062  (0.043) | −0.061  (0.042) |
| *B* |  |  | −0.001  (0.022) | −0.001  (0.021) |  |  |  | −0.046  (0.029) | −0.047^*^  (0.028) |
| *C* |  |  | 0.021  (0.022) | 0.021  (0.021) |  |  |  | −0.035  (0.029) | −0.036  (0.029) |
| *y* |  |  | 0.083^***^  (0.026) | 0.082^***^  (0.026) |  |  |  | −0.137  (0.087) | −0.136  (0.085) |
| *z* |  |  | 0.121^***^  (0.042) | 0.122^***^  (0.041) |  |  |  | −0.147  (0.163) | −0.144  (0.160) |
| Intern experience |  |  |  | 0.121^***^  (0.018) |  |  |  |  | 0.107^***^  (0.025) |
| Female |  |  |  | 0.066^***^  (0.020) |  |  |  |  | −0.123^***^  (0.027) |
| Constant | 0.827^***^  (0.009) | 0.647^***^  (0.030) | 0.698^***^  (0.044) | 0.601^***^  (0.046) |  | 0.940^***^  (0.012) | 1.046^***^  (0.043) | 1.375^***^  (0.298) | 1.417^***^  (0.293) |
| Observations | 1584 | 1584 | 1584 | 1584 |  | 984 | 984 | 984 | 984 |
| *N* | 88 | 88 | 88 | 88 |  | 55 | 55 | 55 | 55 |
| Pseudo *R*^2^ | 0.023 | 0.042 | 0.051 | 0.087 |  | 0.028 | 0.033 | 0.041 | 0.068 |

Robust standard errors are in parentheses. ^***^*p* < 0.01, ^**^*p* < 0.05, ^*^*p* < 0.1. The lower and the upper limits are the minimum and maximum of *α*, respectively.
